# Study on the evolution patterns and predictive modeling of ambient air quality in oasis cities of arid regions: a case study of Urumqi

**DOI:** 10.3389/fpubh.2025.1632713

**Published:** 2025-08-01

**Authors:** Bian Jing, Ren Xiaofeng

**Affiliations:** ^1^College of Education, Qinghai Normal University, Qinghai, Xining, China; ^2^Gansu Qilian Mountain Water Conservation Forest Research Institute, Zhangye, China

**Keywords:** air, Urumqi, air quality—climate interactions, pollution characteristics, air quality

## Abstract

**Background:**

This study investigates the evolution patterns and future trends of ambient air quality in oasis cities within arid regions, with Urumqi as a representative case.

**Methods:**

Utilizing observational data from eight urban monitoring stations, we comprehensively analyze air quality variations and project future scenarios through the Air Quality Index (AQI), Spearman’s rank correlation coefficient, and Grey correlation modeling. Our aim is to elucidate the contributions of atmospheric pollutants to ambient air quality in arid oasis cities.

**Results:**

The results show that: (1) In 2022, Urumqi’s AQI ranged from 24 to 363, with exceed rates of 2.5% for severely polluted weather, 6.15% for heavily polluted conditions, 5.8% for moderate pollution, and 10% for mild pollution. (2) Dust events elevated inhalable particulate matter (PM) concentrations by 5 μg·m^−3^, contributing 6.5% to pollution levels, while the ambient air quality composite index reached 4.45. Dust’s specific contribution to this index was 0.05(1.1%).(3) Meteorological factors—precipitation, wind speed, temperature, and vapor pressure—exhibited significant negative correlations (*p* < 0.05) with PM₂.₅, PM₁₀, SO₂, NO₂, and CO concentrations, but a positive correlation with O₃. Wind speed showed a strong negative association with NO₂ (*p* < 0.01), while temperature and vapor pressure were positively linked to O₃ (*p* < 0.01). (4) The GM (1,1) model demonstrated high predictive accuracy, with errors between 1.0 and 4.2%. Projections indicate a rising trend in ozone concentrations, with the 90th percentile O₃_-8 h_ potentially exceeding 160 μg·m^−3^ by 2025.

**Conclusion:**

These results provide critical insights into the spatiotemporal dynamics of air pollution in Urumqi and its natural drivers, offering a scientific basis for regional air quality management and pollution mitigation strategies.

## 1 Introduction

Ambient air quality is a process in which the concentration of ambient air pollutants produced by people in the process of daily production and life exceeds a certain limit at a specific time, which affects physical comfort, damages physical health, and then affects the economic society and human living environment. The main pollutants affecting the ambient air quality are SO_2_, NOx, CO, O_3_ and particulate matter (PM10, PM_2.5_) (1, 2). Ambient air quality is an important health indicator that reflects the living conditions and quality of life of the people. Its quality directly affects the investment environment of the city. Therefore, it is widely concerned by the government and the people (3). With the rapid development of social economy, motor vehicles and industrial emissions are increasing, and the air quality of urban environment is becoming worse and worse. Air pollution has become a prominent urban environmental problem. From a health point of view, the latest research results of the World Health Organization indicate that PM_2.5_ is still the most serious air pollutant affecting human health. 5 μg·m^−3^ is the concentration value that current research believes can better protect the health of residents. For every 10 μg·m^−3^ increase in PM_2.5_ concentration, the non-accidental mortality rate will increase by 8%. To this end, the World Health Organization issued the “Global Air Quality Guidelines” in 2021, tightening the average annual concentration of PM_2.5_ from 10 to 5 μg·m^−3^ to better protect human health.

As a developing country, China has won the battle against poverty. After the war, improving the ambient air quality is a key task in our new era, and the task is very heavy. At present, China has placed the management of ambient air quality projects in a very prominent national strategic position, formulated and issued national standards and policy regulations for ambient air quality, “Ambient Air Quality Standards (GB3095-2012),” “Environmental Air Pollution Prevention Law of the People’s Republic of China,” “Ambient Air Quality Index (AQI) Technical Regulations (Trial),” and so on. By integrating resources and strengthening regional linkage and interoperability, various regions have formed a regional collaborative pollution control model. A large number of studies have been devoted to exploring the variation of PM and gaseous pollutant concentrations under meteorological conditions such as dust storms and haze (4, 5). The results showed that the cumulative days of moderate and above pollution in prefecture-level cities in Northwest China from 2015 to 2020 were 4,599 days. The days of moderate and above pollution in each province (region) were: Xinjiang (4,627 days) > Shaanxi (1829 days) > Gansu (1,046 days) > Ningxia (569 days) > Qinghai (263 days) (6).

As the core area of the “Belt and Road” strategy, Xinjiang shoulders the dual mission of ensuring national energy security and the stable development of the autonomous region (7). With the rapid development of economy and the acceleration of urbanization, the problem of atmospheric particulate pollution is becoming more and more serious (8, 9). As the capital of Xinjiang Uygur Autonomous Region, Urumqi is located in the northern foot of Tianshan Mountains and the southern margin of Junggar Basin. It is a typical valley city. The special geographical location, the long heating period in winter and the rapid urbanization process make Urumqi face severe air pollution problems (10, 11). The two deserts are the main sources of sand and dust in Asia. Heavy pollution often causes low visibility and foggy weather. The mixing effect of blowing sand and anthropogenic aerosols has a great influence on the concentration level of particulate matter in Urumqi (12). Since 2016, Urumqi ‘s air quality has improved due to the adjustment of energy structure, but it is still at a low level in the air quality ranking of key cities in China due to the impact of urban expansion and the increase in the number of motor vehicles (13). The population of Urumqi is about 4.5 million, and the number of motor vehicles has been growing rapidly (6, 14). By the end of 2022, the number of motor vehicles in Urumqi was 1.5 million, an increase of 4% over 2021 (15, 16). From the perspective of emission source structure analysis, coal and petrochemical industries, energy exploitation and industry are relatively concentrated (17). In 2020, coal accounts for 62% of energy consumption in Urumqi, while the proportion in eastern China is 1.9% (14), especially during the winter heating period (October to April) (14). Soot pollution is the main pollution in the heating period, while dust pollution is the main pollution in the non-heating period (18). In addition, heavy industries such as chemical industry and non-ferrous metal smelting in Urumqi are densely distributed (9). The separation of the Tianshan Mountains in central Xinjiang has formed two different types of pollution areas in southern and northern Xinjiang. In spring, the temperature rises rapidly and the windy weather occurs frequently, which is a windy and sandy season. The air pollution mainly comes from the respirable particulate matter released by the dust, and the air pollution in southern Xinjiang is more than that in northern Xinjiang. In winter, the atmospheric stratification is relatively stable and prone to inversion, which is not conducive to the diffusion of pollutants. In addition, urban emissions are aggravated, and air pollution in northern Xinjiang is more than that in southern Xinjiang (19). As mentioned above, Urumqi is located in the northern foot of the Central Tianshan Mountains and the southern margin of the Junggar Basin. The northern part is surrounded by the Gurbantunggut Desert and the Taklimakan Desert in the southwest. The climate is dry, and it is often affected by dust storms in spring and autumn, which seriously restricts the ventilation of air pollutants (9, 17, 20, 21). In summary, human factors and weather and terrain conditions are the main factors affecting the quality of air quality in Urumqi.

Urumqi is not only an important node city in the core area of the Belt and Road, but also the largest city in Central Asia and its surrounding areas within 1,500 kilometers. This paper chooses Urumqi as the research object, which has the representational and radiation of the node cities in the western arid area of “the Belt and Road.” Systematic analysis of the evolution law, characteristics, existing problems and causes of ambient air quality in Urumqi, and exploration of a series of scientific, efficient, sustainable and worthy of promotion of environmental air pollution control methods can enrich the connotation of government environmental governance macro-control to a certain extent. The research results are expected to provide reference for the management of ambient air quality in oasis cities in arid areas, and also provide scientific basis for local governments to formulate more scientific and reasonable policies.

## 2 Data and methods

### 2.1 Study area

Urumqi (42^°^45^′^ ~ 45^°^20^′^ N, 86^°^37^′^ ~ 88^°^58^′^E) is one of the densely populated cities in Xinjiang. The western, eastern and southern parts are bordered by the Tianshan Mountains, and the northern part is the southern margin of the Junggar Basin. It is an arid oasis city under the mountain basin system. The temperate continental arid climate is the hottest in July and August within 1 year, with an average temperature of 25.7°C. January is the coldest month, with an average temperature of −15.2°C. In winter, it is controlled by cold high pressure, and often has calm wind weather. The atmospheric stratification is stable and the inversion layer is thick, which leads to the difficulty of pollutant diffusion. As of 2022, the city has 7 districts and 1 county, with a total area of 13,800 km^2^, a resident population of 4.08 million, and a vehicle ownership of 130.98 × 10^4^ vehicles. The central urban area includes the new urban area (326km^2^), Shuimogou District (277.56 km^2^), Tianshan District (245 km^2^), and Shayibake District (328 km^2^). The long heating period, low wind speed, strong air stability and easy to produce inversion layer in winter make the horizontal and vertical diffusion capacity of atmospheric pollutants in Urumqi very low, which provides conditions for the accumulation of atmospheric pollutants.

**Figure 1:**
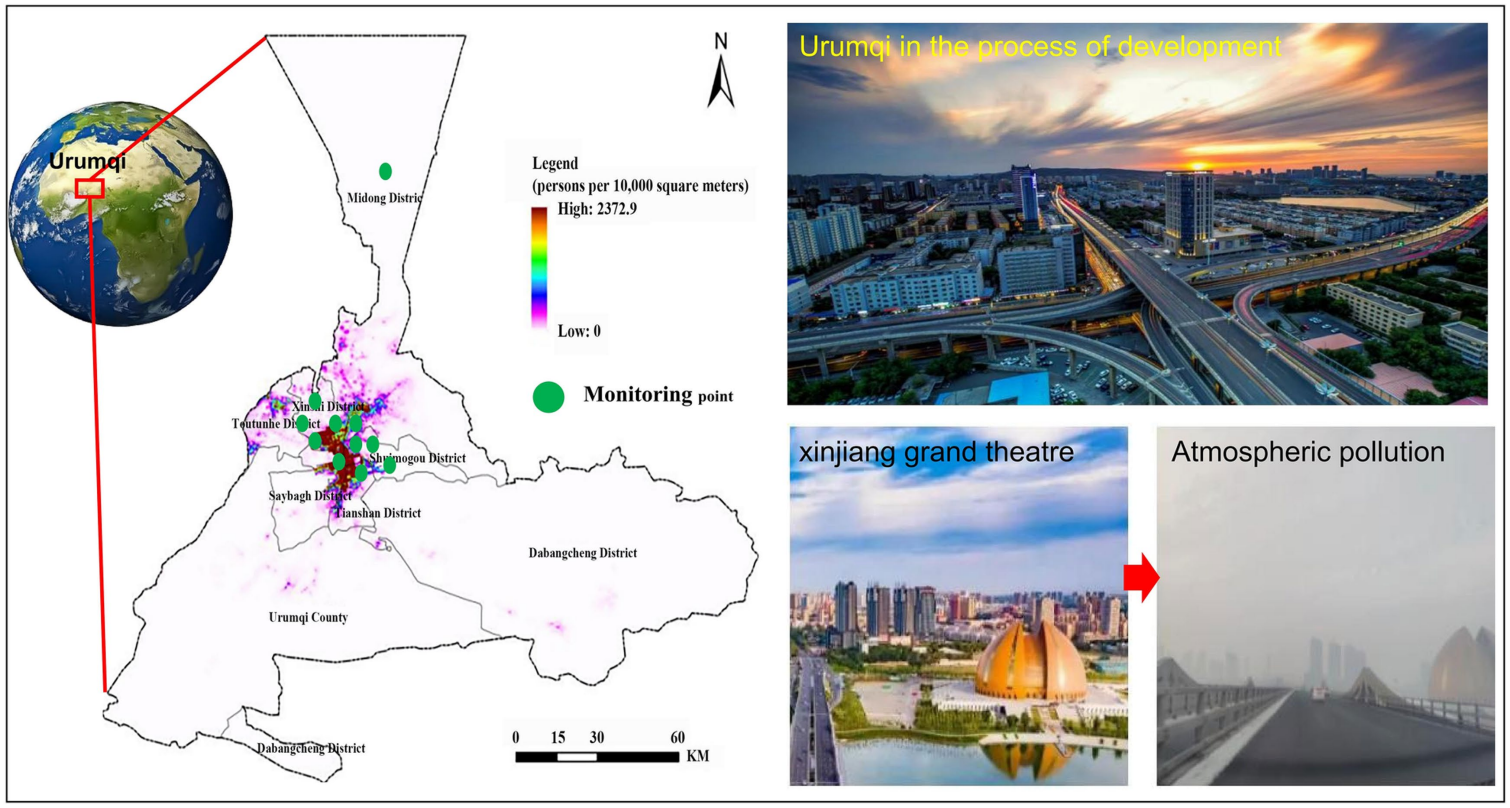
Study area and monitoring sites.

The monitoring data of atmospheric pollutants are derived from the hourly concentration data of 10 fixed air quality monitoring stations of training base, Dabancheng District Environmental Protection Bureau, Railway Bureau, monitoring station, toll station, Hongguang Mountain, Hot Spring Campus of Xinjiang Normal University, 31 Middle School, Great Green Valley and Midong District Environmental Protection Bureau. The daily average concentration values of PM_10_, CO, NO_2_, SO_2_, and PM_2.5_ are used, and the daily maximum 8-h sliding average concentration of O_3_ is used. The heating period in Urumqi is from October 10 to April 10 of the next year, and the non-heating season is from April 11 to October 9. The daily monitoring period is 00:00–24:00, and the missing hourly monitoring data are removed (Figure 1).

### 2.2 Methods

(1) AQI air quality index

The air quality index is a standard used to describe the quality of ambient air quality. It is a dimensionless number. The higher the AQI index, the more serious the pollution of air quality in the environment, and the greater the harm to the environment and human health. In the calculation, the maximum value of air quality index in 6 kinds of pollutants (SO_2_, NO_2_, PM10, PM_2.5_, CO, O_3_) was taken. The calculation formula is as follows Equation (1):


(1) $${\text{IAQI}}_{P}=\frac{\left({\text{IAQI}}_{\mathrm{Hi}}-{\text{IAQI}}_{L0})(C_{P}-BP_{L0}\right)}{\left({\mathrm{BP}}_{\mathrm{Hi}}-{\mathrm{BP}}_{L0}\right)}+{\text{IAQI}}_{L0}$$

In the formula, IAQI_P_ is the air quality score of the pollution project P; C_P_ is the concentration of pollution item P; BP*_Hi_* is the upper limit of the corresponding standard concentration; BP_L0_ is the lower limit of the corresponding standard concentration; IAQI*_Hi_* is the air quality sub-index corresponding to BP*_Hi_*; IAQI*_L0_* is the air quality sub-index corresponding to BP*_L0_*.

The air quality index (AQI) is calculated by comparing the individual pollutants of SO_2_, NO_2_, PM10, PM_2.5_, CO, and O_3_. When the AQI is greater than 50, the pollutant with the largest IAQI is the primary pollutant. The ambient air quality sub-index of the pollutant project P is calculated according to Equation (2):


(2) $$\mathrm{AQI}=\max\{{\text{IAQI}}_{1},{\text{IAQI}}_{2},{\text{IAQI}}_{3},\cdots ,{\text{IAQI}}_{n}\}$$

In the formula: IAQI is the air quality sub-index, n is the pollutant item (Table 1).

(2) Spearman rank correlation coefficient method

**Table 1:** AQI level situation.

Numerical value	Air quality index level
0 < AQI < 50	Level 1 (excellent)
51 < AQI < 100	Level 2 (good)
101 < AQI < 150	Level 3 (mild pollution)
151 < AQI < 200	Level 4 (moderate pollution)
201 < AQI < 300	Level 5 (heavy pollution)
AQI > 300	Level 6 (severe pollution)

Heavy pollution days refer to Grade 5 (severe pollution) and Grade 6 (severe pollution) weather.

As shown in Equation 3, the Spearman rank correlation coefficient method is used to calculate the rank correlation coefficient rs. The absolute value of rs is compared with the critical value in the Spearman Rank Correlation coefficient statistical table. If |rs| ≥ W_P_, it indicates that the trend change of each pollution index is significant. If |rs| < W_P_, it indicates that the index changes smoothly during the study period, and the change trend is not statistically significant. If rs is positive, it indicates that the variable change is an upward trend, and if rs is negative, it indicates that the variable change shows a downward trend.


(3) $$r_{s}=1-\frac{6\sum_{i=n}^{n}{\left(x_{i}-y_{i}\right)}^{2}}{n(n^{2}-1)}$$

In the formula, n is the number of time periods of each pollution index; r*_s_* is the rank correlation coefficient; x*_i_* is the time series of the annual average value of each pollution index from small to large. *y_i_* is the time series of annual order.

(3) Gray correlation analysis model

Grey correlation analysis is a multi-factor statistical method, which is an important branch of Grey system theory. The basic idea is to judge whether the relationship between different sequences is close according to the geometric shape of the sequence curve. Suppose there are several data sequences X*_0_*, X*_1_*,…, X*_n_* and Y*_0_*, Y*_1_*,…, Y*_n_*; among them, all the research object sequence Y*_i_* is the reference sequence, and the rest is the comparison sequence X*_i_*. The calculation formula is as follows Equations (4–9):

(1) Find the initial image value of each sequence

(4) $$X_{i}^{\prime }=\frac{X_{i}}{x_{i}(1)}=(x_{i}^{\prime }(1),x_{i}^{\prime }(2),\cdots ,x_{i}^{\prime }(n)),i=0,1,\cdots ,m$$

In the formula: x*_i_* represents the *i* the original data sequence; x*_i_* (1) represents the first data point of the sequence x*_i_*, that is, the value of x*_i_* at time point t = 1; x*_i_* “represents the sequence after standardization (initial image transformation), which is defined as xi divided by its first data point xi (1); x*_i_* ′ (j) denotes the value of the normalized i-the sequence at time point j, i.e., x*_i_* ′ (j) = x*_i_* (j)/x*_i_* (1), j = 1,2,…, n.

(2) Find the absolute value sequence of the difference between the classifications corresponding to the initial image values of each sequence

(5) $$\Delta_{i}(k)=(x_{0}^{\prime }(k)-x_{i}^{\prime }(k))$$

(6) $$\Delta_{i}=(\Delta_{i}(1),\Delta_{i}(2),\cdots ,\Delta_{i}(n)),i=1,2,\cdots ,m$$

In the formula: Δ*_i_*(k) denotes the absolute value sequence of the difference between the ith comparison sequence and the reference sequence X*_0_*; δi represents a complete sequence of absolute values of differences, including the absolute values of k = 1, 2,…, n at all-time points.

(3) Calculate the maximum and minimum values of the absolute value sequence, respectively

(7) $$\left\{ \begin{array}{l} M=\max_{i}\max_{k}\Delta_{i}(k)\\ m=\min_{i}\min_{k}\Delta_{i}(k) \end{array}\right.$$

where, k = 1, 2,…, n.

(4) Calculate the correlation number

(8) $$\gamma_{0i}(k)=\frac{m+\mathrm{\xi M}}{\Delta_{i}(k)+\mathrm{\xi M}},\xi \in (0,1)$$

(5) Solve the average value of the correlation number, that is, the grey correlation degree

(9) $$\gamma_{0i}=\frac{1}{n}\sum_{k=1}^{n}\gamma_{0i}(k)$$

## 3 Results

### 3.1 Air environmental quality change rule and its influencing factors

#### 3.1.1 Air environmental quality change rule

**Figure 2:**
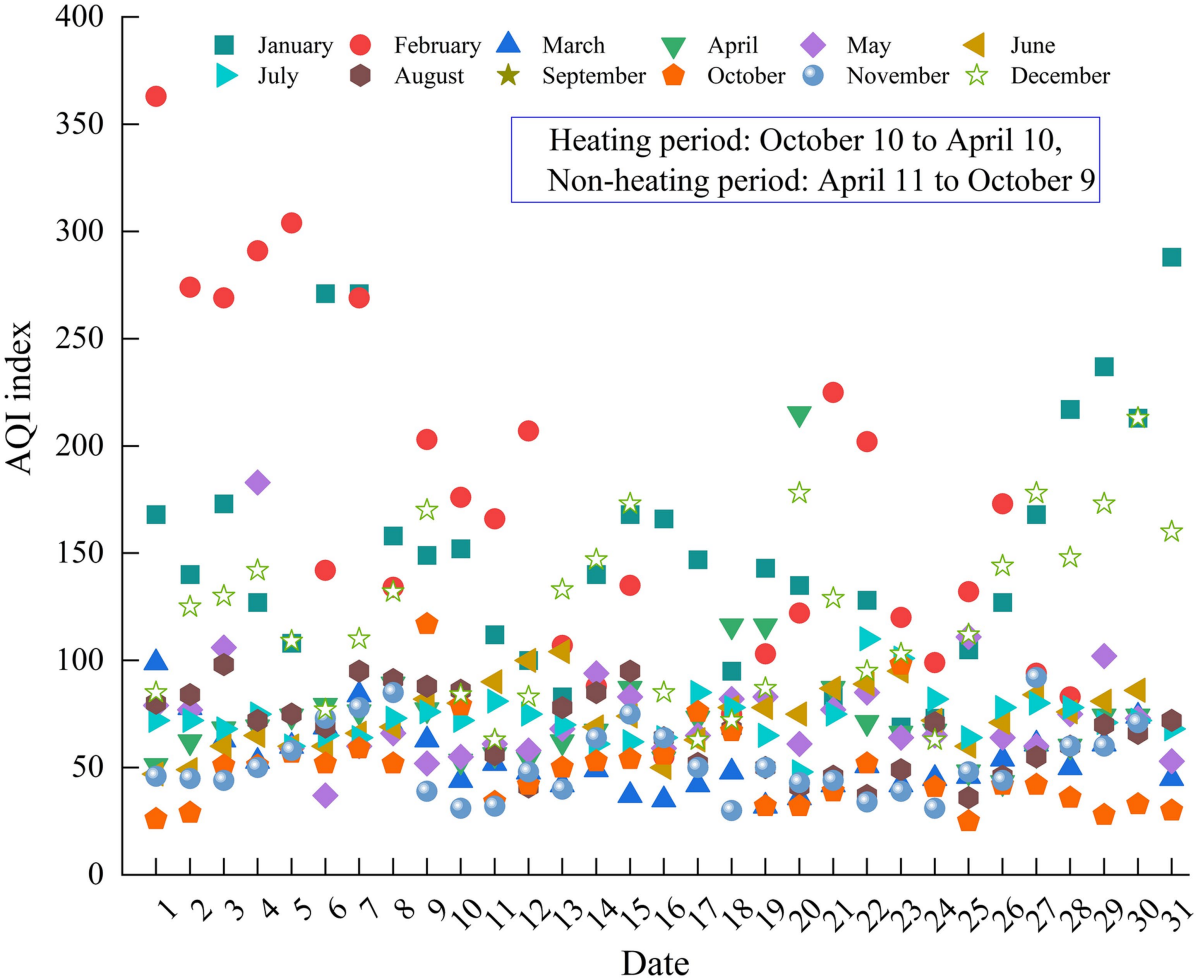
The scatter plot of AQI index in Urumqi.

As shown in Figure 2, the number of effective monitoring days in Urumqi in 2022 is 365 days, the city’s air quality AQI index is between 24 and 363, and the number of excellent days is 285 days, which is 10 days less than that in 2021 (hereinafter referred to as year-on-year). Among them, the number of excellent days was 72 days, the number of good days was 213 days, and the air quality compliance rate was 78.1%, a year-on-year decline of 2.7%. The number of days in which air quality was polluted to varying degrees was 80 days, an increase of 10 days; mild pollution for 45 days, an increase of 3 days; moderate pollution for 17 days, an increase of 2 days; 16 days of heavy pollution, an increase of 4 days; severe pollution for 2 days, an increase of 1 day. In 2022, the over-standard rate of AQI in severe polluted weather in Urumqi was 2.5%, which was concentrated in January, February and early December. The over-standard rate of AQI in severe pollution weather was 6.15%, and the over-standard rate of AQI in moderate pollution weather was 5.8%, which was concentrated in early March and mid-December. The over-standard rate of AQI in lightly polluted weather was 10%, which was concentrated in late March and November. The proportion of weather with good and excellent AQI was 58.35 and 19.7%, respectively, concentrated in April–October. According to the seasonal study, the proportion of excellent days from high to low is: summer (91.21%) > spring (72.33%) > autumn (70.12%) > winter (60%). According to the degree of pollution, it can be divided into four grades, which are mild pollution (14.21%) > moderate pollution (7%) > severe pollution (4.25%) > severe pollution (2.02%). Among them, mild, moderate and severe pollution weather mainly occur in winter, accounting for 26, 10 and 4.23%, respectively. Severe pollution weather mainly occurred in spring, accounting for 7.02%.

#### 3.1.2 Effects of pollution days and dust days on air quality

**Figure 3:**
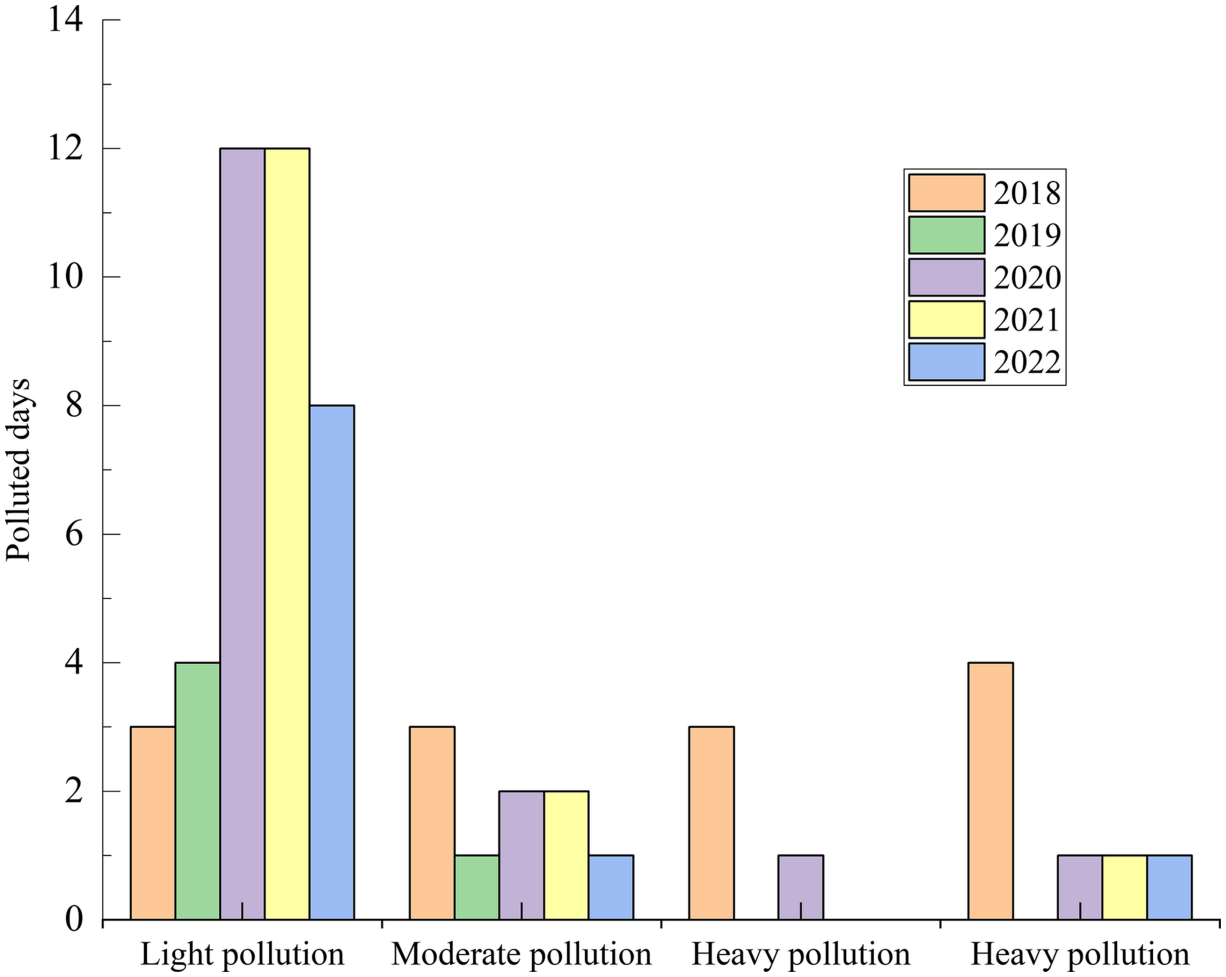
Days of pollution at all levels caused by sand and dust in 2018–2022.

Considering the concentration of pollutants in the air and the impact on health, when AQI exceeds 100, it is defined as a pollution day (22). When the hourly concentration of PM_10_ is greater than or equal to 150 μg.m^−3^ and PM_2.5_/PM10 is less than or equal to 0.30 (PM_2.5_/PM10 is less than or equal to 0.40 in winter), it is defined as a dust-affected day (23). When the hourly concentration of PM_10_ in cities or stations near the dust source area lasts more than 600 μg.m^−3^ for 2 h, or more than 1,000 μg.m^−3^ for 1 h, the monitoring data of particulate matter in the source area and downstream cities in the area affected by dust weather can be deducted. As shown in Figure 3, the number of days affected by sand and dust in Urumqi in 2022 was 22 days, and the primary pollutant was all PM_10_. Among them, the number of days causing various types of pollution totaled 10 days, and the number of days that did not significantly affect air quality totaled 12 days (24). Dust significantly increased the average annual concentration of PM_10_ while affecting the number of good days. Dust days were mainly concentrated in March, April, May, July, August, September, October and November. The number of days of dust weather in the above months was 1, 6, 6, 1, 1, 2, 4, and 1 day, respectively. The daily average concentration of PM_10_ in dust weather in 2022 was 153 μg.m^−3^, and the maximum value was 360 μg.m^−3^ (occurred on April 20), which was consistent with the larger dust monitoring data in April. The maximum value was 2.4 times of the national standard limit (150 μg.m^−3^), and the minimum value was 65 μg.m^−3^, which was 0.4 times of the national standard limit. The annual dust caused serious pollution for 1 day, moderate pollution for 1 day, mild pollution for 8 days, and the remaining 12 days did not cause pollution due to the short duration of dust, and the air quality was good.

**Figure 4:**
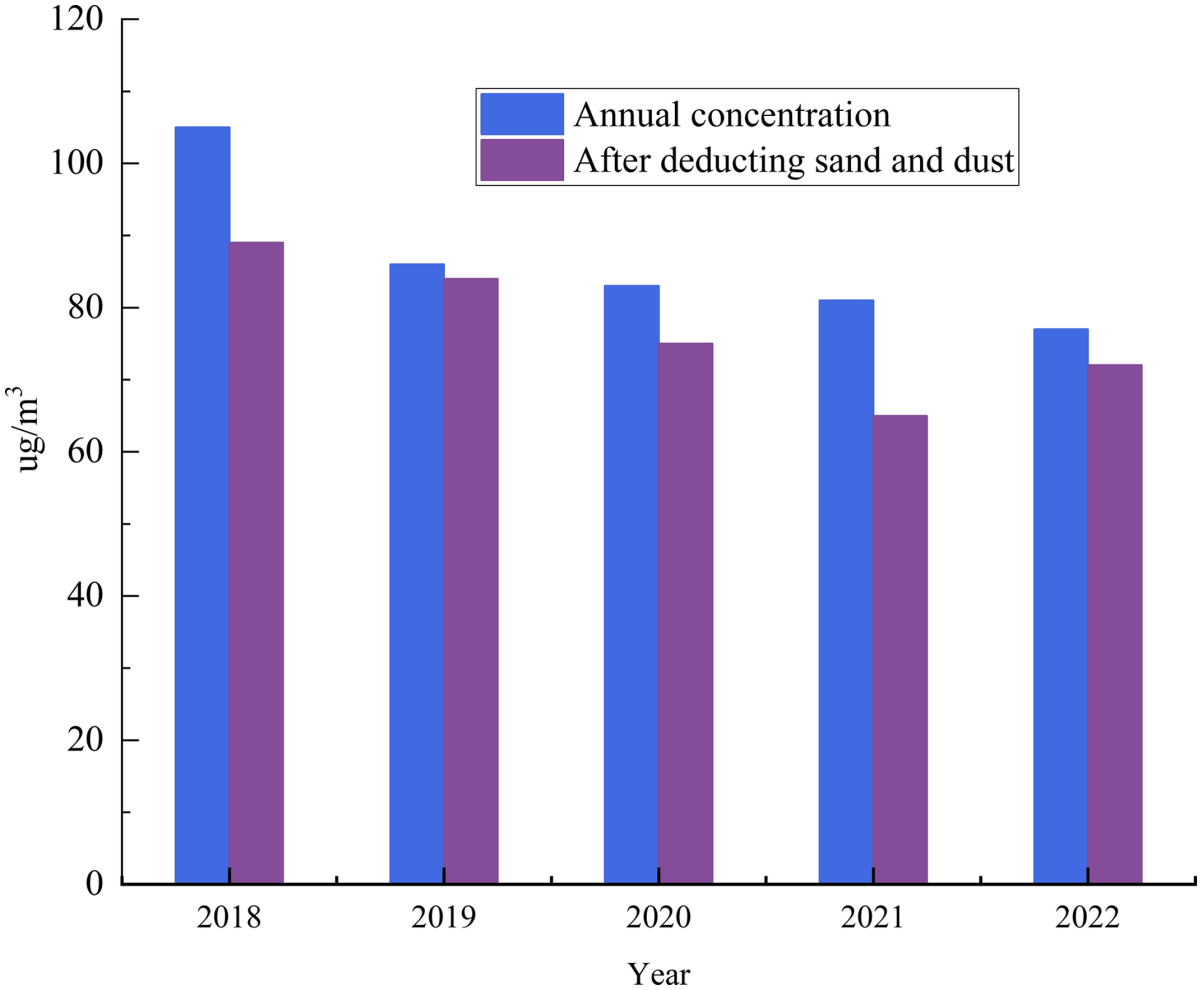
The impact of dust on the annual average concentration of urban PM_10_ in 2018–2022.

As shown in Figure 4, the average annual concentration of PM_10_ in the past 5 years showed a decreasing trend, but the dust days had a serious negative impact on the ambient air quality, and the dust days significantly increased the annual PM_10_ concentration level. In 2021, dust increased PM_10_ concentration by 16 μg·m^−3^, with a contribution rate of 19.8%. In 2022, dust increased the PM_10_ concentration by 5 μg·m^−3^, the contribution rate was 6.5%, and the comprehensive index of ambient air quality was 4.45. Among them, the contribution value of dust to the comprehensive index was 0.05, and the contribution rate was 1.1%. Compared with 2021, although there are three more days of dust in 2022, the duration of dust is shorter and the level of dust is lower, which does not significantly affect the ambient air quality, so the contribution rate is significantly reduced.

#### 3.1.3 Influence of meteorological elements on air quality

Table 2 is the correlation coefficient of daily average concentration of six pollutants in Urumqi. There was a significant correlation between the six pollutants at the 0.01 level. There was a significant positive correlation between PM_2.5_ and CO concentration, and the correlation coefficient was 0.991. There was a significant positive correlation between PM_10_ concentration and NO_2_ concentration, and the correlation coefficient was 0.978. There was a significant positive correlation between NO_2_ concentration and SO_2_ concentration, and the correlation coefficient was 0.981. O_3_ concentration was significantly negatively correlated with PM_2.5_, PM10, SO_2_, NO_2_, and CO concentration, and the negative correlation with CO concentration was the most significant.

**Table 2:** The scatter plot of AQI in Urumqi in 2022.

Contaminant	PM_2.5_	PM_10_	NO_2_	SO_2_	CO	O_3_
PM_2.5_	1					
PM_10_	0.990^**^	1				
NO_2_	0.971^**^	0.978^**^	1			
SO_2_	0.938^**^	0.958^**^	0.981^**^	1		
CO	0.991^**^	0.974^**^	0.970^**^	0.950^**^	1	
O_3_	−0.779^**^	−0.748^**^	−0.754^**^	−0.760^**^	−0.831^**^	1

** is significantly correlated at the 0.01 level.

As shown in Table 3, precipitation, wind speed, air temperature and water pressure were significantly negatively correlated with PM_2.5_, PM10, SO_2_, NO_2_, and CO concentrations, and positively correlated with O_3_ concentration. Among them, precipitation was significantly correlated with SO_2_ and NO_2_ concentrations at the 0.05 level, wind speed was relatively negatively correlated with NO_2_ concentration at the 0.01 level, and air temperature and water pressure were relatively positively correlated with O_3_ at the 0.01 level. The average pressure and relative humidity were positively correlated with PM_2.5_, PM10, SO_2_, NO_2_, and CO concentrations, and negatively correlated with O_3_ concentration. The pressure was significantly negatively correlated with O_3_ concentration at 0.01 level, and the relative humidity was positively correlated with CO concentration at 0.01 level. The main reason is that the weather is cold during the heating period, the temperature drops, the heating increases the coal supply, and some coal-fired heating points emit more NO_2_, CO, and PM_2.5_ pollutants. During the daytime, the temperature gradually increases, and the primary pollutant NO_2_ undergoes a chemical reaction to generate the secondary pollutant O_3_ under the sun’s irradiation. At night, NO and other pollutants react with O_3_ to produce NO_2_. The wind speed is conducive to the diffusion and dilution of NO_2_, CO, and PM_2.5_, reducing their concentration and contributing to the generation of O_3_. Relative humidity is negatively correlated with SO_2_, NO_2_, and O_3_, and positively correlated with PM_2.5_ and CO, indicating that relative humidity affects the absorption rate of some pollutants. There is almost no liquid precipitation during the heating period. Therefore, the dilution effect on pollutants is limited. With the increase of relative humidity, the absorption rate of particulate matter in the atmosphere is increased, and it is positively correlated with relative humidity.

**Table 3:** The scatter plot of AQI in Urumqi in 2022.

Meteorological element	Precipitation (0.1 mm)	Average air pressure (0.1 hPa)	Average wind speed (0.1 m•s^−1^)	Mean air temperature (0.1°C)	Average water pressure (0.1 hPa)	Mean relative humidity (1%)
PM_2.5_	−0.545	0.887^**^	−0.825^**^	−0.914^**^	−0.791^**^	0.871^**^
PM_10_	−0.590	0.845^**^	−0.811^**^	−0.869^**^	−0.758^**^	0.820^**^
NO_2_	−0.625^*^	0.838^**^	−0.849^**^	−0.887^**^	−0.760^**^	0.800^**^
SO_2_	−0.690^*^	0.838^**^	−0.838^**^	−0.847^**^	−0.780^**^	0.752^**^
CO	−0.579	0.902^**^	−0.848^**^	−0.939^**^	−0.830^**^	0.899^**^
O_3_	0.522	−0.939^**^	0.750^**^	0.937^**^	0.980^**^	−0.824^**^

** is significantly correlated at the 0.01 level. * is significantly correlated at the 0.5 level.

### 3.2 The change rule of primary pollutant concentration

PM_2.5_ and PM_10_ are the primary pollutants of ambient air quality. SO_2_, NO_2_, and O_3_ are the primary pollutants for fewer days, and CO is not the primary pollutant. In 2022, the average concentration of pollutants in the 10 evaluation sites in the city was compared with that in the same period last year (Table 4). The average annual concentration of SO_2_ was 7 μg.m^−3^, which was the same as that in the same period last year. The concentration of NO_2_ was 31 μg.m^−3^, decreased by 18.4%. The concentration of PM_10_ was 72 μg.m^−3^, decreased by 2.7%. The concentration of PM_2.5_ was 42 μg.m^−3^, increased by 5.0%. The 95th percentile of daily average CO concentration was 1.8 mg.m^−3^, which was flat year-on-year. The 90th percentile of daily average O_3_ concentration was 136 μg.m^−3^, increased by 1.5%. In 2022, the composite index was 4.43, down 2.8% year-on-year.

**Table 4:** The average concentration of pollutants in the city compared with the same period last year.

Year	Air quality compliance rate	SO_2_ (μg/m^3^)	NO_2_ (μg/m^3^)	PM_10_ (μg/m^3^)	PM_2.5_ (μg/m^3^)	CO (mg/m^3^)	O_3–8 h_ (μg/m^3^)	Comprehensive pollution index
2021	80.80%	7	38	74	40	1.8	134	4.56
2022	78.10%	7	31	72	42	1.8	136	4.43
Contrast	−3.30%	–	−18.40%	−2.70%	+5.00%	–	+1.50%	−2.85

**Figure 5:**
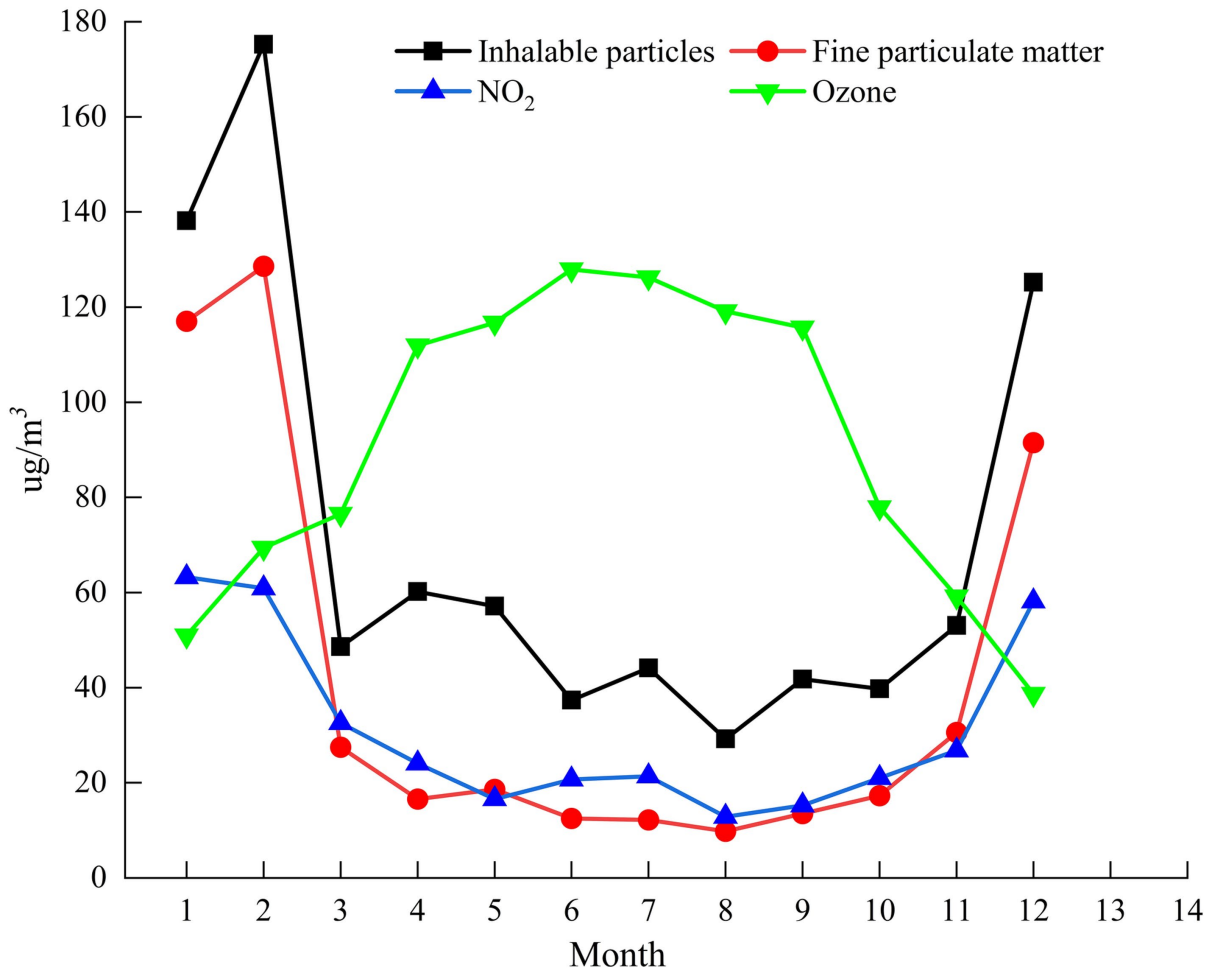
PM10, PM_2.5,_ NO_2,_ O_3_ concentrations were analyzed.

The monthly average concentration changes of PM10, PM_2.5_, NO_2_, and O_3_ in Urumqi in 2022 are shown in Figure 5. PM10, PM_2.5_, NO_2_, and O_3_ showed obvious seasonal changes. The monthly concentration curves of PM10, PM_2.5_, and NO_2_ generally showed a “U-shaped” change, with January and December as the annual peak, May to July as the relative valley, January at the peak and then decreasing month by month, showing an upward trend in October. It can be seen from the monthly average change curve of O_3_ that O_3_ shows an “inverted U-shaped” change, and has obvious seasonal changes. The valley value is from December to January of the next year, and reaches the peak in June, indicating that the change of pollution concentration is directly related to the influence of weather changes, which is consistent with the trend of most northern cities. The highest monthly average concentration of O_3_ was in summer July (120 μg.m^−3^), mainly due to the strong sunshine in summer, which effectively promoted the photochemical reaction of nitrogen oxides and volatile organic compounds in the air, resulting in an increase in O_3_ concentration. The NO_2_ in the atmosphere mainly comes from the energy industry, traffic source and industrial source, which is related to the dense distribution of Urumqi petrochemical enterprises and economic development zones. The monthly average concentrations of PM_10_ and PM_2.5_ were basically the same during the year, peaked in February, then gradually decreased, decreased to a relatively low point in August, and began to rise from August, November to January of the following year was the relative high value of the whole year. The above two pollutants show obvious seasonality. The concentration value is low in summer and high in winter. In addition to the increase of industrial production in the off-peak season and the increase of coal consumption for heating, it may also be related to the better growth of plants in summer and the absorption of polluted gases by green value.

**Figure 6:**
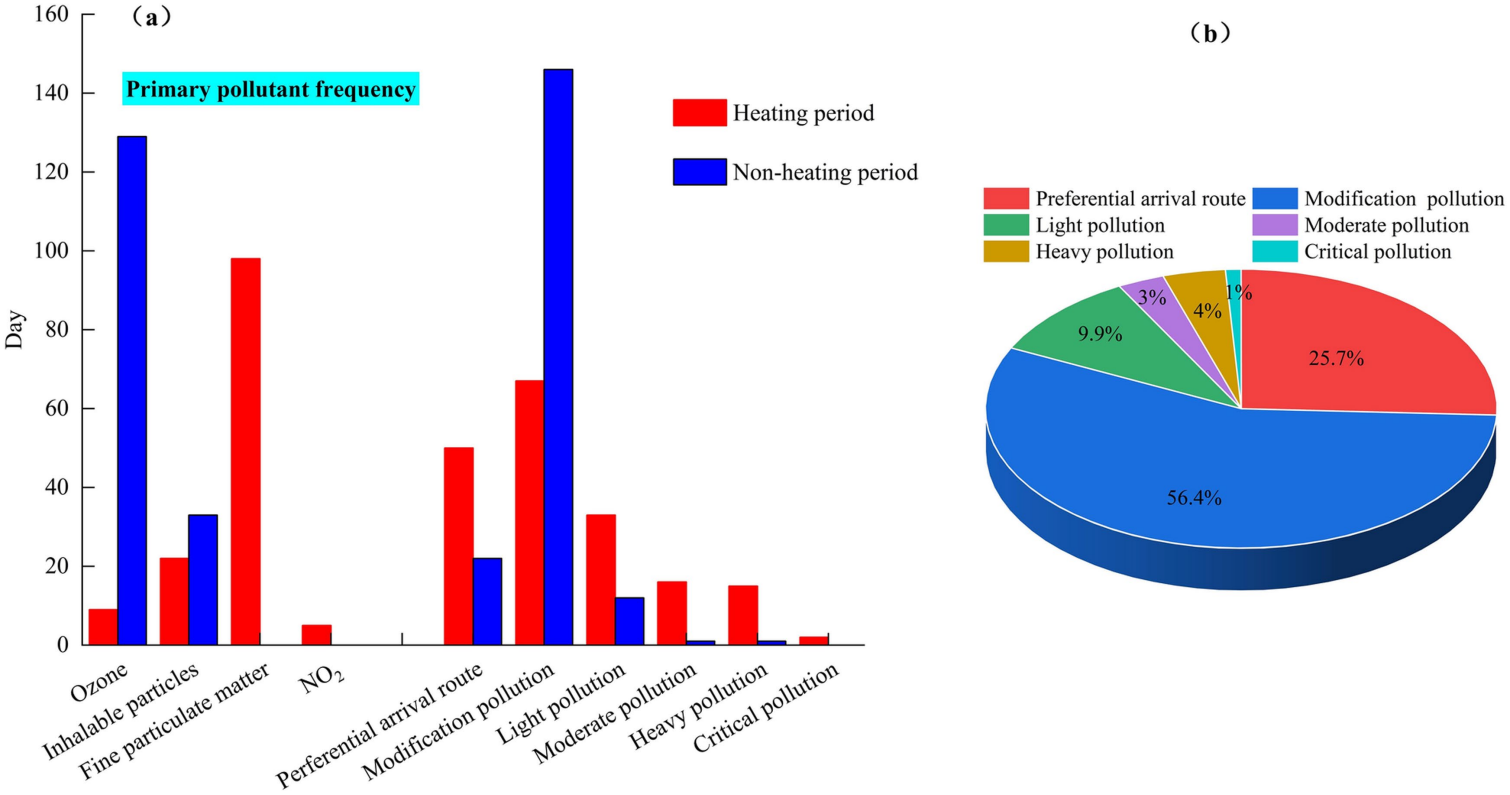
Frequency statistics of primary pollutants in heating and non-heating periods **(a)** and proportion of days at all levels of air quality **(b)**.

In 2022, the city’s PM10 and PM_2.5_, O_3_, and NO_2_ as the primary pollutants were 55, 98, 138, and 5 days, accounting for 15, 27, 37, and 1%, respectively. The heating period is mainly PM_10_ and PM_2.5_, respectively 98 days and 22 days; the non-heating period is mainly O_3_ and PM_10_, which are 129 and 33 days, respectively, as shown in Figure 6. The above pollutant weather is affected by natural factors such as wind and sand transit, dust pollution in winter and spring, and human factors such as a large amount of coal burning in the heating period. In summer, it is in the non-heating period, with less emission sources such as coal combustion. In addition, the meteorological conditions are good, and pollutants are easy to diffuse. Secondly, it is also closely related to the special geographical location of Urumqi and the topographic conditions of the huge mountain basin system. The region is surrounded by mountains on three sides, and the terrain is tilted from south to north, showing a horn shape as a whole. In summer and autumn, the valley wind is obvious, the troposphere is thick, and the convective motion is frequent, which is conducive to the diffusion of pollutants. In spring and winter, when the cold air from the Junggar Basin moves southward, it is blocked by the Tianshan Mountains and turns downward. It accumulates in Urumqi at the foot of the mountain, forming a thick inversion layer. The atmospheric stratification is stable, which is not conducive to the vertical diffusion of pollutants. At the same time, wind speed and wind direction affect the degree of pollutant diffusion and agglomeration.

**Figure 7:**
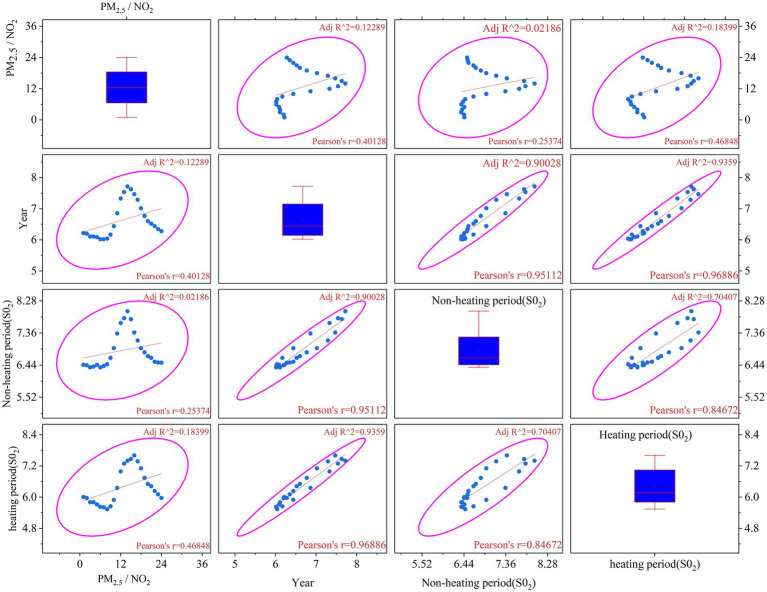
Scatter plot of SO_2_ concentration in heating period and non-heating period.

As shown in Figure 7, the blue scatter+fitting curve represents the linear trend between variables, the pink confidence ellipse represents the confidence interval of the data distribution (usually 95%), and the blue box plot represents the quartile and outliers of the data distribution. R^2^ represents the goodness of fit of the regression model, 0 ~ 1, the closer to 1, the stronger the explanatory power. (Pearson’s r) represents the linear correlation between variables (−1 ~ 1, the greater the absolute value, the stronger the correlation). In 2022, the average annual concentration of SO_2_ in Urumqi was 7 μg·m^−3^, reaching the standard. The daily average range was 4 ~ 14 μg·m^−3^, all reaching the standard, and the maximum concentration peak appeared in the heating period. By comparing the non-heating period and the heating period (lower right corner subgraph), *R*^2^ = 0.70407, the fitting is better, indicating that there is a strong correlation between the two. Pearson’s *r* = 0.84672, showing a high positive correlation, reflecting that SO_2_ emission sources (such as industrial or coal-fired) exist throughout the year, but the increase during the heating period is more obvious. The box plot shows that the median difference is about 2.5 times, highlighting the superimposed effect of coal-fired heating. It can be seen that the high concentration of SO_2_ in Urumqi mainly comes from the emission of local heating coal.

In summary, the strong correlation between SO_2_ in the heating period and the year (*r* = 0.96886) proves that the control policy is effective, and it is recommended to expand the coverage of “coal to gas.” The moderate correlation between PM_2.5_/NO_2_ and industrial SO_2_ (*r* = 0.46848) indicates that it is necessary to strengthen the ultra-low emission transformation of steel and cement industries, as well as the treatment of motor vehicle exhaust (especially diesel vehicles).

**Figure 8:**
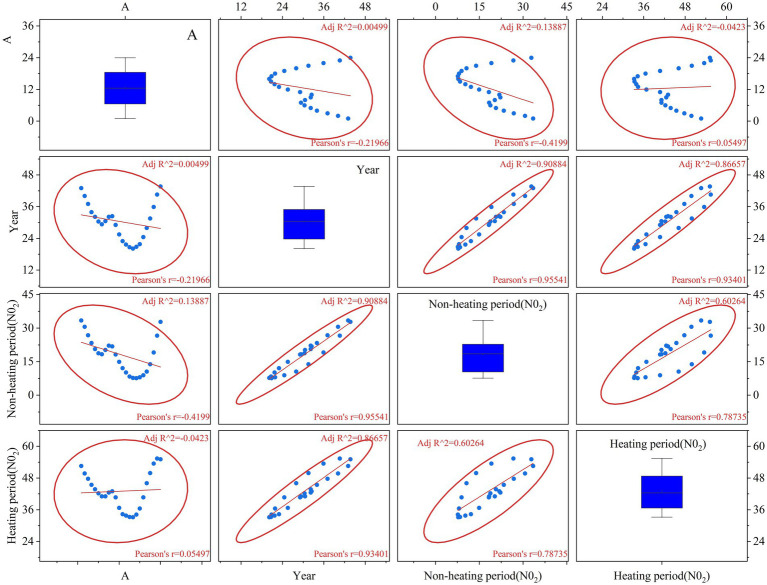
Scatter plot of NO_2_ concentration in heating period and non-heating period.

Similarly, the variation of NO_2_ was analyzed. As shown in Figure 8, the NO_2_ concentration in the heating period was significantly higher than that in the non-heating period. Compared with before 2022, the concentration of NO_2_ in the heating period showed a strong positive correlation with the policy, *r* = 0.93401, *R*^2^ = 0.98657, which reflected the obvious effect of coal-fired heating transformation (such as “coal to gas”). The average annual decrease of NO_2_ in the heating period was 5.3%, and the slope was −2.4 μg.m^−3^/year. With the development of industrial control measures, NO_2_ in the non-heating period showed a sub-strong decline compared with that before 2022, *r* = 0.95541, *R*^2^ = 0.90884. This shows that industrial denitrification (such as SCR technology) has a significant effect. In conclusion, NO_2_ showed a high correlation between heating period and non-heating period, *r* = 0.78735, *R*^2^ = 0.60264, which revealed the annual impact of common emission sources (such as power plants), but the concentration in heating period was 62% higher than that in non-heating period. From the perspective of the discreteness of NO_2_ in the heating period, the IQR value reaches 24-48 μg.m^−3^, reflecting the lack of heating stability, such as the lag of boiler start-stop regulation.

### 3.3 Air environmental quality assessment and future prediction analysis

#### 3.3.1 Spearman rank correlation coefficient method for air quality evaluation

**Figure 9:**
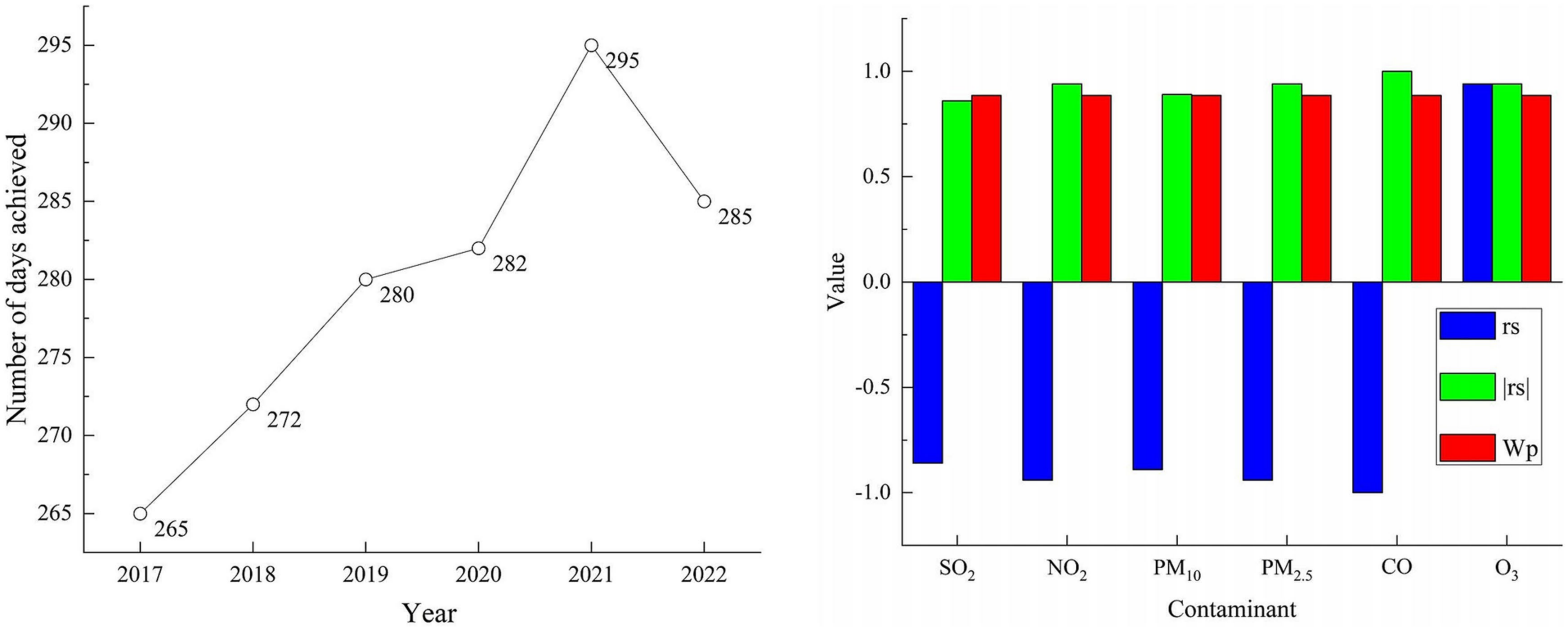
rs values of atmospheric pollution factors in Urumqi from 2017 to 2022.

Because the critical value n of the rank correlation coefficient is at least 6, the data of 2017 are added to calculate the rank correlation coefficient of six pollution factors such as PM_10_ and PM_2.5_ in the atmospheric environment of Urumqi from 2017 to 2022. The results are shown in Figure 9.

The |rs| value of SO_2_ is 0.86 W_P_ value, the |rs| value of PM_10_ is 0.89 > W_P_ value, the |rs| value of PM_2.5_ is 0.94 > W_P_ value, the |rs| value of CO is 1 > W_P_ value, and the |rs| value of O_3_ is 0.94 > W_P_ value, that is, the annual change trend of SO_2_ is not significant, and the annual change trend of the remaining five pollution factors is significant. Among them, the rs of O_3_ is positive, indicating that the annual change of its index shows an upward trend; the rs values of NO_2_, PM_10_, PM_2.5_ and CO were negative, indicating that the annual changes of these indicators showed a downward trend. In the past 6 years, the atmospheric environment in Urumqi has improved, and the city’s air pollution control measures have achieved certain results.

#### 3.3.2 Influencing factors of air environmental quality assessment

##### 3.3.2.1 Gray correlation analysis of national economy and industrial pollutant emissions

The emissions of industrial waste gas pollutants SO_2_, NO compounds and particulate matter were selected. In order to measure and compare the degree and order of correlation between pollutants and Urumqi’s gross domestic product (GDP), the gross domestic product (GDP) of Urumqi from 2016 to 2022 was used as the parent sequence, and various types of exhaust gas emissions were used as sub-sequences. The Grey correlation analysis model method was used to establish the correlation model, as shown in Table 5.

**Table 5:** Grey correlation degree between GDP and industrial pollutant emissions in Urumqi.

	GDP (billion yuan)	Wastewater pollutant emissions				Emissions of waste gas pollutants		
		Chemistry oxygen demand	Ammonia nitrogen	Total nitrogen	Total phosphorus	SO_2_	Nitrogen oxide chemicals	PM
Degree of association (R_0i_)	1	0.9414	0.9061	0.9165	0.8976	0.6518	0.5805	0.6013

The correlation degree between industrial waste gas pollutant emissions and gross domestic product (GDP) from large to small is SO_2_, particulate matter and NO, that is, the correlation degree between sulfur dioxide emissions and gross domestic product (GDP) is the highest, and the correlation degree between nitrogen oxide emissions and gross domestic product (GDP) is the lowest. The Grey correlation model shows that the indicators affecting the air environmental quality pollution in Urumqi are mainly concentrated in energy consumption and industrial pollution emissions. First of all, industrial pollution sources are one of the important pollution sources in cities. SO_2_ emissions from industrial waste gas and soot emissions have the greatest impact on SO_2_ in the air. The dust in industrial waste gas has a great influence on NO_2_ in the air, which seriously affects the air quality of Urumqi city. Secondly, economic growth and the improvement of living standards inevitably drive energy consumption and become an important source of environmental pollution. According to the analysis results, percapita living consumption has a great impact on SO_2_, PM10, NO_2_ and annual air quality compliance rate. In a word, the energy consumption brought by life and economic development affects the air quality of Urumqi to a great extent. The total number of cars in the city cannot be ignored, which is also one of the important factors affecting the compliance rate of NO_2_ and air quality. The increase of urban resident population also has a certain impact on air pollution concentration and compliance rate. The increase of urban heating area has a great effect on the elimination of soot, and the correlation coefficient with the air quality compliance rate ranks in the top five. Urban collective heating has a great contribution to improving the overall air quality compliance rate.

##### 3.3.2.2 Grey correlation analysis of national economy and domestic pollutant emissions

Similarly, the Grey correlation analysis model is used to establish the correlation between the emission of domestic pollutants and the gross domestic product (GDP) of Urumqi. The emissions of sulfur dioxide, nitrogen oxides and particulate matter from domestic waste gas pollutants were selected. The results show that the correlation degree between the emission of domestic source exhaust gas pollutants and gross domestic product (GDP) is NO compounds, SO_2_ and particulate matter in descending order, that is, the correlation degree between the emission of nitrogen oxides in domestic source exhaust gas pollutants and gross domestic product (GDP) is the highest, and the correlation procedure between particulate matter emission and gross domestic product (GDP) is the lowest (Table 6).

**Table 6:** Gray correlation degree between GDP and domestic pollutant emissions in Urumqi.

	GDP (billion yuan)	Wastewater pollutant emissions				Emissions of waste gas pollutants		
		Chemistry oxygen demand	Ammonia nitrogen	Total nitrogen	Total phosphorus	SO_2_	Nitrogen oxide chemicals	Particulate matter
Degree of association(R_0i_)	1	0.6466	0.8806	0.7598	0.7980	0.8875	0.9094	0.7220

#### 3.3.3 Prediction of air environmental quality

The annual concentration data of main pollutants and the comprehensive index of ambient air quality in Urumqi from 2015 to 2022 were arranged in chronological order, and the data sets of SO_2_, NO_2_, PM10, PM_2.5_, CO, and O_3_ were used as input conditions, respectively. Among them, SO_2_, NO_2_, PM10, and PM_2.5_ used the annual average concentration data of Urumqi, carbon monoxide used the 95th percentile concentration data, and ozone used the 95th percentile concentration data. The model formula parameter development coefficient a value and the Grey action u value of each input term are calculated respectively, as shown in Table 7.

**Table 7:** Model parameters (u, a) output.

Model parameter	SO_2_	NO_2_	PM_10_	PM_2.5_	CO	O_3_	Aggregative index number
u	15.4506	53.4565	113.9449	78.7184	4.0511	98.6042	7.0383
a	0.1191	0.0682	0.0529	0.105	0.1154	−0.0472	0.0667

By calculating the mean square error ratio (c) and small error probability (p) of each input term, the applicability accuracy range of the established model is judged by the residual error. It is verified that the accuracy of the GM (1,1) model constructed by each input data set is first-level (good), as shown in Table 8, indicating that the model is available and has predictive significance and ability.

**Table 8:** Model accuracy parameter (p, c) output and model applicability accuracy determination.

Model parameter	SO_2_	NO_2_	PM10	PM_2.5_	CO	O_3_	Aggregative index number
p	1	1	1	1	1	1	1
c	0.0831	0.0823	0.1663	0.0578	0.005	0.2194	0.0003
Prediction model Accuracy level	First-order	First-order	First-order	First-order	First-order	First-order	First-order

**Figure 10:**
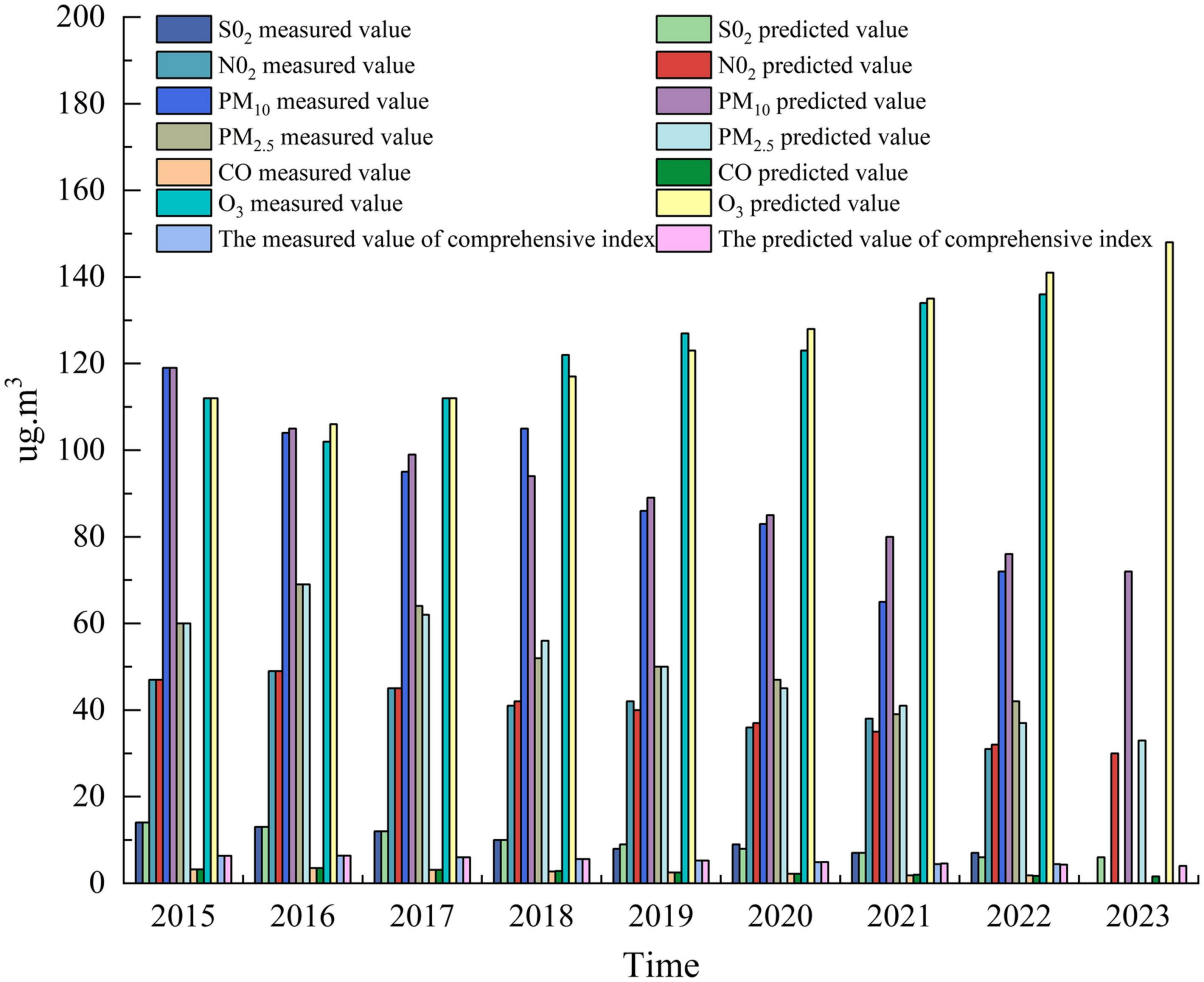
Prediction results of ambient air quality GM (1,1) model.

The results of GM (1,1) model for PM_10_ prediction show that the error between the predicted value and the measured value of the model is between 1.0 and 4.2%, and the anti-validation effect is good. The anti-validation and prediction results are shown in Figure 10. According to the prediction results, the average annual concentration of PM_10_ in Urumqi will reach 69 μg·m^−3^ in 2024, which meets the requirements of the secondary standard of “Ambient Air Quality Standard” (70 μg·m^−3^). The annual average concentration of PM_2.5_ will meet the secondary standard (35 μg·m^−3^) of ‘Ambient Air Quality Standard’ around 2023. According to the prediction results of the model, there will still be a risk of continuous increase of O_3_ concentration in Urumqi in the next 5 years, and it is possible that the 90th percentile concentration of O_3–8 h_ will exceed 160 μg·m^−3^ in 2025, so it is urgent to formulate effective policies.

The prediction of GM (1,1) model is based on the preliminary judgment of the actual data change trend in recent years. The prediction results only reflect the subsequent impact of the current changes, and the realization of the specific results and the arrival of the goals still depend on the intensity, intensity and sustainability of air pollution prevention and control and other related pollution prevention and control work of the government and environmental authorities. In order to achieve the predicted downward trend results of the model, the management department must scientifically formulate the next environmental air quality control objectives based on the actual situation. In addition, we should focus on solving the key pollution problems, such as the problem of increasing ozone year by year and the serious pollution in winter. Scientific research and demonstration should be carried out to find out the causes and mechanisms of pollution, the possibility of improvement and specific improvement measures. Based on the results of scientific research and the experience of advanced treatment in China, a special plan for air pollution prevention and control of key issues was formulated and implemented to promote the continuous improvement of the atmospheric environment.

## 4 Suggestions

### 4.1 Actively adjust the urban energy structure and promote the use of new energy

The long-term development of Urumqi has formed an industrial structure biased toward traditional heavy industries such as petroleum, chemical industry and steel. Compared with developed cities dominated by modern advanced manufacturing, the emission of pollutants from traditional heavy industries has a far-reaching impact on the quality of urban atmospheric environment. Adjust the industrial structure, from the traditional petroleum, chemical, iron and steel industry structure type to modern advanced manufacturing, green manufacturing, strengthen the wind energy, hydrogen energy, photovoltaic and other clean energy, renewable energy equipment construction layout and supporting power grid construction, further improve the utilization rate of clean energy, reduce the city’s urban energy (heating, vehicle) in the proportion of fossil (coal, natural gas) energy.

### 4.2 Strictly implement the project access management, promote industrial optimization and upgrading

We will resolutely curb the blind development of high-energy-consuming and high-emission projects, implement the hard constraints of ecological protection red line, environmental quality bottom line, and resource utilization on the line into the environmental management and control unit, and increase the proportion of green and low-carbon industries in the city’s total economic output. We should actively promote the optimization and upgrading of existing industries, carry out performance classification and implement the strictest system management for enterprises in key industries such as steel, cement, petrochemical, casting, printing and surface spraying.

### 4.3 Strengthen the control of motor vehicles and oil products to reduce VOCs emissions

Reducing VOCs emissions has become an important means to control the increase of ozone concentration. In order to curb the rapid rise of ozone, it is necessary to strictly implement various measures for motor vehicle pollution prevention and control while doing a good job in the control of industrial enterprises involving VOCs emissions. Strictly implement the requirements of motor vehicle emissions and oil control at all stages of the country, increase the elimination of old machinery and motor vehicles, and encourage green travel. Taking vehicles in the public domain as the starting point, new or updated urban public transport, urban logistics distribution, light postal express delivery, taxis, official vehicles, light sanitation vehicles and other priority use of new energy vehicles, and introduce relevant policies to encourage private vehicles to use new energy vehicles, reduce vehicle exhaust emissions, thereby reducing the concentration of ozone VOCs in the ambient air.

## 5 Conclusion

(1) The air quality in Urumqi has improved to a certain extent. The number of excellent days is 72 days, the number of good days is 213 days, and the air quality compliance rate is 78.1%. The proportion of excellent days from high to low was summer (91.21%) > spring (72.33%) > autumn (70.12%) > winter (60%). The degree of pollution can be divided into four levels, accounting for mild pollution (14.21%) > moderate pollution (7%) > severe pollution (4.25%) > severe pollution (2.02%). Mild, moderate and severe pollution weather mainly occurred in winter, accounting for 26, 10, and 4.23%, respectively. Severe pollution weather mainly occurred in spring, accounting for 7.02%.
(2) In 2022, the number of days affected by sand and dust in Urumqi was 22 days, and the primary pollutant was all PM_10_. The daily average concentration of inhalable particulate matter (PM_10_) in dust weather was 153 μg·m^−3^, the maximum value was 360 μg·m^−3^, the maximum value was 2.4 times of the national standard limit (150 μg·m^−3^), and the minimum value was 65 μg·m^−3^, which was 0.4 times of the national standard limit. Actively adjust the urban energy structure and promote the use of new energy; strictly implement project access management and promote industrial optimization and upgrading; strengthen the control of motor vehicles and oil products to reduce VOCs emissions.
(3) In 2022, the average concentration of pollutants in the 10 evaluation points in the city was compared with the same period last year. It was found that the average annual concentration of SO_2_ was 7 μg·m^−3^, which was flat year-on-year; the concentration of NO_2_ was 31 μg·m^−3^, decreased by 18.4%. The concentration of PM10 was 72 μg·m^−3^, decreased by 2.7%. The concentration of PM_2.5_ was 42 μg·m^−3^, which increased by 5.0%. The 95th percentile of daily average CO concentration was 1.8 mg·m^−3^, which was flat year-on-year. The 90th percentile of daily average O_3_ concentration was 136 μg·m^−3^, increased by 1.5%. In 2022, the composite index was 4.43, down 2.8% year-on-year.

## Data Availability

The datasets presented in this study can be found in online repositories. The names of the repository/repositories and accession number(s) can be found in the article/Supplementary material.
